# CCIW: Cover-Concealed Image Watermarking for Dual Protection of Privacy and Copyright

**DOI:** 10.3390/e27121198

**Published:** 2025-11-26

**Authors:** Ruiping Li, Si Wang, Ming Li, Hua Ren

**Affiliations:** 1 Information Construction and Management Office, Henan Normal University, Xinxiang 453007, China; 2 School of Computer and Information Engineering, Henan Normal University, Xinxiang 453007, China; wangsi@stu.htu.edu.cn (S.W.); liming@htu.edu.cn (M.L.); renhua@htu.edu.cn (H.R.); 3 Henan Key Laboratory of Educational Artificial Intelligence and Personalized Learning, Xinxiang 453007, China

**Keywords:** image watermarking, privacy protection, copyright protection, adversarial example, DNN

## Abstract

Traditional image watermarking technology focuses on the robustness and imperceptibility of the copyright information embedded in the cover image. However, in addition to copyright theft, the cover images stored and transmitted in the open network environment is facing the threat of being identified and retrieved by deep neural network (DNN) with malicious purpose, which is a new privacy threat. Therefore, it is essential to protect the copyright and the privacy of cover image simultaneously. In this paper, a novel cover-concealed image watermarking (CCIW) is proposed, which combines conditional generative adversarial networks with channel attention mechanisms to generate adversarial examples of the cover image containing invisible copyright information. This method can effectively prevent privacy leakage and copyright infringement simultaneously, since the cover image cannot be collected and processed by DNNs without permission, and the embedded copyright information is hardly to be removed. The experimental results show that the proposed method achieved a success rate of adversarial attack over 98% on the Caltech256 dataset, and the generated adversarial examples have good image quality. The accuracy of copyright information extraction is close to 100%, and it also exhibits good robustness in different noise environments.

## 1. Introduction

With the widespread application of digital images in social networks, the security of image data has attracted increasing attention. The security threats faced by images not only involve the copyright protection of images but also include privacy risks. In particular, with the development of deep learning technology [1], Deep Neural Networks (DNN) have greatly improved the performance of tasks such as image classification, face recognition, and image generation. However, this also provides adversaries with more advanced attack methods, that is, attackers can use DNN classifiers to screen out target images from social platforms and carry out infringing acts such as tampering with and stealing these images. Consequently, the problem of malicious search and identification of images based on DNN in the big data environment has emerged, posing new challenges to image privacy protection.

Meanwhile, research on adversarial examples has revealed the vulnerability of deep learning models, which are often maliciously exploited to launch attacks and deceive neural networks. Szegedy et al. [2] found that the input and output of DNN learning are largely discontinuous. Adding small and imperceptible perturbations to natural images can cause a well-trained model to output a different classification of the original image with high confidence. Such images generated by adding perturbations are called adversarial examples. Subsequently, Goodfellow et al. [3] proposed the Fast Gradient Sign Method (FGSM) for studying adversarial examples, and argued that the vulnerability of neural networks to adversarial perturbations stems from the linear nature of high-dimensional networks, rather than the non-linearity of high-dimensional networks as traditionally believed. However, the adversarial examples generated by this method have relatively weak attack effects and are easily detected. To improve the attack performance of adversarial examples, Carlini and Wagner [4] proposed the C&W method, which generates adversarial examples by optimizing the loss function. Compared with FGSM, it is more accurate and can produce stronger attack effects.

With the in-depth research on adversarial examples, their application in the field of privacy protection has gradually gained attention. Researchers have found that adversarial examples can effectively utilize sensitive regions in images to achieve image privacy protection, making these regions “invisible” to DNNs and thus preventing data leakage [5,6]. Su et al. [7] used optimization algorithms to identify more sensitive regions in original images and embedded perturbations into these regions to generate adversarial examples; experimental results verified that this method effectively improves attack performance. Zhu et al. [8] discovered that adding perturbations to the S channel of HSV images can enhance attack success rates while maintaining good image visual quality. For this reason, they proposed the Stochastic Perturbation Gradient Descent (SPGD) adversarial example method. Meanwhile, some studies restore images by reversing the cover images of adversarial examples. For instance, Yin et al. [9] proposed a reversible attack method based on adversarial perturbations in the YUV color space and reversible data hiding. Experiments show that this method can recover the original image without distortion while achieving good attack capability and image visual quality. Huang et al. [10] proposed an Invertible Reversible Adversarial Examples (I-RAE) scheme based on Invertible Neural Networks (INN) for reversible adversarial examples. This scheme combines the Black-Box Attack Flow (BAFLOW) and a perturbation hiding network, successfully realizing the image recovery of adversarial examples; however, this method still suffers from a relatively low attack success rate. Current research has shown that image classification and recognition systems based on deep learning exhibit significant vulnerabilities when facing adversarial examples. Nevertheless, existing adversarial example technologies can only address privacy protection issues in a single manner and cannot fully meet comprehensive image security requirements, including copyright protection.

In terms of copyright protection, robust watermarking [11] technology has been extensively studied. Traditional robust watermarking techniques embed watermarks into various transform domains of the carrier to enhance resistance against image attacks. For example, Fu et al. [12] leveraged the high robustness of Fractional Orthogonal Moments (FoOM) and the geometric attack resistance of Zernike Moments and Pseudo-Zernike Moments (ZM/PZM). By combining these two properties, they proposed a robust watermarking method that is resistant to both geometric attacks and conventional noise attacks. To further improve the overall performance of watermarks, many machine learning algorithms have been applied in digital watermarking. For instance, Tang et al. [13] proposed a robust digital watermarking algorithm based on Discrete Cosine Transform (DCT) and Support Vector Machine (SVM). In this algorithm, watermarks are embedded using block-based DCT, and SVM is used to correct geometric parameters. With the advent of deep learning, the use of DNN has also become a mainstream direction in digital watermarking research [14,15]. To better ensure the robustness of digital watermarks against JPEG compression, Jia et al. [16] trained an end-to-end network architecture by Mini-Batch of Real and Simulated JPEG Compression (MBRS). This architecture enhances the robustness of the watermarking model by randomly selecting real JPEG, simulated JPEG, and noise-free layers in small batches to serve as the noise layers of the network. Traditional robust watermarking focuses on the robustness and invisibility of watermarks, which can effectively protect image copyright. There are also some new watermarking schemes with multi-functions, e.g., Padhi et al. [17] used perceptual hash as well as cryptographic hash in deep learning-based watermarking technique, and performed image copyright protection and authentication simultaneously. However, the above researches fail to address the privacy threats posed by DNN to massive digital images in open network environments.

In recent years, some studies have attempted to combine robust watermarking technology (which enables copyright protection) with adversarial example technology (which enables privacy protection). This combination protects image copyright by verifying the integrity of hidden information, while using the attack capability of adversarial examples to counter the collection threats from DNN, thereby achieving comprehensive protection of images. For example, Jia et al. [18] proposed a new adversarial watermark perturbation algorithm called Adv-watermark by combining adversarial examples with image watermarking. They used the Basin-Hopping Evolution (BHE) algorithm to optimize the generation of adversarial watermarks and proved that the generated watermarked images have good robustness against various transformation-based defenses. Jiang et al. [19] proposed a method named FAWA (Fast Adversarial Watermarking with Differential Evolution) that uses fast differential evolution technology to quickly generate adversarial watermarks. This method selects the optimal way to embed watermarks into images, while enabling the generated watermarked images to successfully deceive DNN classifiers. Experiments have shown that this method has good attack capability and portability (can be smoothly migrated to another environment). However, the image watermarks generated by the aforementioned combination of robust watermarking and adversarial examples are often visible to the human eye. Although they possess adversarial attack properties, they compromise image quality.

Subsequently, some studies have replaced robust watermarks with invisible binary copyright information. For example, Wang et al. [20] proposed a method Adversarial Data Hiding (ADH) that uses genetic algorithms to explore decision boundaries in a constrained search space and hides data in “sensitive pixels” to generate adversarial examples. However, this method still suffers from issues such as small information hiding capacity and low success rate. To improve the attack success rate and reduce damage to images, Li et al. [21] proposed a method to generate adversarial examples by modifying only one pixel, aiming to achieve both data hiding and adversarial attack functions. Nevertheless, the modified single pixel is significantly visible to the naked eye, and the information hiding capacity is too small to carry sufficient authentication information for protecting the copyright and authenticity of images. Additionally, the attack success rate is insufficient to achieve satisfactory performance in privacy protection. To enhance the efficiency of image copyright protection, Wang et al. [22] designed an adversarial watermark fusion model to efficiently generate invisible adversarial watermarks (IAW). Experimental results show that this method achieves good performance in aspects such as extraction accuracy and attack capability. Although the aforementioned methods that combine information hiding with adversarial examples provide privacy protection and copyright protection capabilities to a certain extent, they still face problems such as poor image quality, low attack success rate, and insufficient robustness in different environments. Furthermore, they perform poorly when confronting more complex attacks.

To address the aforementioned issues, this paper proposes a novel approach of cover-concealed image watermarking (CCIW): embedding copyright information into the host image invisibly in the form of adversarial perturbations to generate adversarial examples with adversarial attack capabilities. By leveraging the global pixel characteristics of the cover image, an identifiable adversarial example model with image copyright protection functionality is designed based on Conditional Generative Adversarial Networks (CGAN) and a channel attention mechanism. This model can not only effectively embed copyright information and ensure the accuracy of its extraction but also exhibit strong robustness to withstand interference in different noise environments. Meanwhile, it maintains high image visual quality and prevents malicious users from using DNN for image collection. Thus, it provides more comprehensive protection for image privacy and copyright protection in network environments.

The contributions of this paper can be summarized as follows:

(1) A new model is proposed to combine digital watermarking technology with adversarial examples based on CGAN and channel attention mechanism, utilizing the invisibility and attack capability of adversarial perturbations to embed copyright information in images.

(2) This approach can not only prevent malicious users from collecting images using DNNs, but also realize the verification and protection of copyright without affecting the visual quality of images.

(3) Experiments and analyses confirm the effectiveness of the proposed method. It not only achieves high adversarial attack performance, but also ensures good image quality, and can effectively resist noise interference in different network environments.

## 2. Preliminary Knowledge

### 2.1. Generative Adversarial Network

The emergence of Generative Adversarial Network (GAN) has brought great convenience to image generation and processing, and has also attracted significant attention from both industry and academia. A GAN can be viewed as a zero-sum game, consisting of a generator (G) and a discriminator (D). The generator is like a “counterfeiting gang” that attempts to produce and use counterfeit money, while the discriminator is like a “police officer who detects counterfeit money”. The generator tries to deceive the discriminator, while the discriminator strives not to be deceived by the generator. The model undergoes alternating optimization training, where both components are improved. Eventually, a generator with significantly enhanced performance is obtained—one that the discriminator cannot distinguish from real data. The final state achieved is called Nash equilibrium.

In the GAN structure, the goal of the generator is to learn the distribution of training data so as to generate more realistic data samples. Typically, it takes a random noise vector as input, and through a series of neural network transformations, maps the noise to the generated sample space. The loss function of the generator is shown in Equation (1):

(1) $$L_{G}=E_{z\sim p_{z}(z)}[\log(1-D(G(z)))],$$

where D(G(z)) denotes the probability that the discriminator considers the generated sample to be real. $z\sim p_{z}(z)$ represents sampling from the noise distribution. Since the generator aims to generate more realistic images, a smaller value of this loss indicates a higher quality of the generated samples.

The goal of the discriminator is to distinguish whether the input sample comes from the real data distribution or the fake data generated by the generator. It is essentially a binary classifier, and its output is the probability that the sample is real. Its loss function is shown in Equation (2):

(2) $$L_{D}=E_{x\sim p_{data}(x)}[\log D(x)]+E_{z\sim p_{z}(z)}[\log(1-D(G(z)))],$$

where D(x) denotes the probability that the discriminator outputs a real data sample as real, D(G(z)) represents the probability that the discriminator outputs a fake data sample (generated by the generator) as real, and $x\sim p_{data}(x)$ indicates sampling from the real data distribution. Since the discriminator aims to determine whether the input sample is real with a higher probability, maximizing this loss is conducive to improving the discriminator’s judgment ability.

The overall objective of GAN can be expressed as a minimax optimization problem, as shown in Equation (3).

(3) $$\underset{G}{\min}\;\underset{D}{\max}\left\{\underset{x∼p_{data}(x)}{E}\left[\log D(x)\right]+\underset{z∼p_{z}(z)}{E}\left[\log(1-D(G(z))\right]\right\}$$

Subsequently, Mirza et al. [23] improved GAN by introducing certain constrained conditional information into the original model, and proposed CGAN. This network uses additional information to control the output, making the generation results of the generator more accurate. For example, in image translation tasks, the conditional information can be the target image, enabling the generator to learn features and convert the input image into the target image.

### 2.2. Attention Mechanism Based on Deep Learning

The visual attention mechanism is a unique neural signal processing mechanism of human vision in the brain. When faced with objects containing large amounts of information, it can avoid interference from massive information and select important information. The attention mechanism based on deep learning draws on the working principle of the visual attention mechanism, thereby identifying the key targets for solving problems. The attention mechanism is mainly classified into three types: hard attention mechanism [24], self-attention mechanism [25], and soft attention mechanism [26].

The hard attention mechanism selects a specific subset of input data, rather than calculating continuous weights and ignoring other parts. Due to its non-differentiable nature, it is usually associated with reinforcement learning. In the process of adversarial image generation, it is necessary to retain as much of the cover image information as possible, i.e., information cannot be ignored, as this would lead to image distortion. Therefore, we do not consider the application of the hard attention mechanism. The self-attention mechanism captures the correlations between image elements. For embedding data to generate adversarial images, attention should focus more on the overall information of the image. Therefore, we prioritize the soft attention mechanism. The soft attention mechanism calculates weights for each part of the input data separately, which are used to represent the degree of attention to different parts.

The soft attention mechanism can be further divided into spatial attention and channel attention. Spatial attention assigns weights in the spatial dimension to emphasize important spatial locations; however, this selective attention may overlook some local information that is less prominent but still important. Additionally, Yu [27] utilized spatial attention to implement information hiding, and the results showed that the regions with low attention sensitivity were not the complex texture or edge regions that are relatively safe for information hiding. Therefore, the performance improvement of spatial attention for generating adversarial functional images by embedding data is limited. Channel attention calculates the weight relationship between channels through global pooling and fully connected layers. It can selectively enhance the channels important for information hiding, suppress unimportant channels, and at the same time reduce noise interference, thereby improving the robustness of the generated images. Tan et al. [28] used channel attention and GAN to realize image information hiding, which not only improved the quality of generated images but also increased the capacity of embedded information. By comparing with other channel attention methods, they proved that the channel attention method proposed in their paper, which integrates max-pooling and average-pooling, achieved more significant performance improvement. Thus, in the model designed to generate adversarial images, we adopt the channel attention mechanism to enhance the image generation performance.

In the attention structure, first, the input feature $F\in R^{M\times H\times W}$ is subjected to max-pooling and average-pooling respectively. Max-pooling is more sensitive to the texture feature information of the image and selects the maximum value $f_{max}\in R^{M\times 1}$ from each channel; average-pooling is more sensitive to the background information of the image and extracts the average value $f_{avg}\in R^{M\times 1}$ from each channel. The $f_{max}$ and $f_{avg}$ of the n-th element are calculated by the following Equations (4) and (5):

(4) $$f_{avg}^{n}=\frac{1}{H\times W}\sum_{i=1}^{H}\sum_{j=1}^{W}F_{i,j}^{n},$$

(5) $$f_{\max}^{n}=\max(\sum_{i=1}^{H}\sum_{j=1}^{W}F_{i,j}^{n}).$$

The obtained $f_{max}$ and $f_{avg}$ are fused through two fully connected layers respectively. Then, the two resulting feature vectors are added element-wise, and the result is normalized using the Sigmoid function, yielding the feature vector $s\in R^{M\times 1}$ weighted in the spatial dimension. This process can be described by the following Equation (6):

(6) $$s=\sigma (W_{2}(\delta (W_{1}f_{\max}))+W_{2}(\delta (W_{1}f_{avg}))),$$

where δ and σ represent the ReLU and Sigmoid activation functions respectively; $W_{1}\in R^{M^{2}/r}$ and $W_{2}\in R^{M^{2}/r}$ are the weight vectors of the two fully connected layers respectively. To balance the model complexity and computational performance, a dimensionality reduction coefficient r is introduced. Next, each element of the obtained channel weight vector s is multiplied by each channel of the input feature vector F to rescale the original features, resulting in the rescaled feature vector $U=[U^{1},U^{2},\ldots ,U^{M}]$, as shown in Equation (7):

(7) $$U^{m}=s^{m}F^{m},m\in [1,M]$$

## 3. Proposed Method

### 3.1. Model Framework

**Figure 1:**
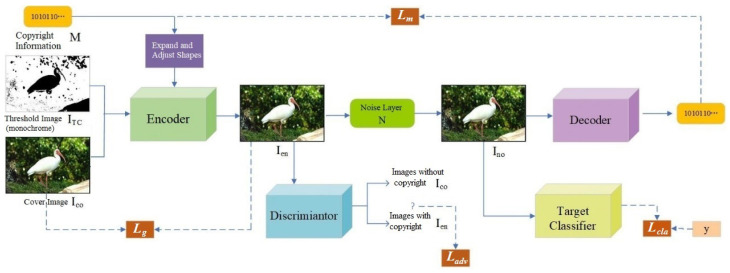
Model Framework of CCIW.

Considering the attack performance of generated adversarial examples and the passive detection performance of robust watermarks, we integrate their advantages based on the CGAN and the channel attention mechanism module. This integration meets the requirements of feature extraction and anti-interference for robust watermarks, while embedding copyright information into partial regions of the cover image to achieve the invisibility of copyright information. As shown in Figure 1, the model framework of the proposed CCIW consists of five components: encoder E, decoder D, noise layer N, discriminator A, and target classifier T.

(1) The encoder E receives the cover image $I_{co}$, threshold image $I_{TC}$, and binary copyright information M, and outputs an adversarial example $I_{en}$ with attack capability.

(2) The noise layer N receives the adversarial example $I_{en}$, randomly selects a type of noise attack to distort the adversarial example, thereby generating a noisy image $I_{no}$.

(3) The decoder D extracts the copyright information $M^{\prime }$ from the noisy adversarial example $I_{no}$.

(4) The discriminator A receives the image $I_{en}$, detects whether the input image is $I_{co}$ or $I_{en}$, and finally returns the detection result.

(5) The target classifier T receives the noise-processed image $I_{no}$ as input and outputs the corresponding classification label.

The processing process of CCIW is as follows. The cover image $I_{co}$ (along with the threshold image $I_{TC}$) and the corresponding copyright information M are encoded by the Encoder, and then the generated encoded image $I_{en}$ is sent to the Discriminator to check if the copyright information can be detected, where the loss function is $L_{adv}$. At the same time, the encoded image $I_{en}$ is compared with the original cover image $I_{co}$ in order to minimize the difference between the two images and preserve high visual quality, where the loss function is $L_{g}$. The encoded image $I_{en}$ containing the copyright information is then processed by the noise layer and generate the noised watermarked image $I_{no}$. To ensure that the watermarked image equips adversarial ability, the noised image $I_{no}$ is further sent to the Target Classifier to predict its label. The difference between the predicted label and the true label is judged by the loss function $L_{cla}$. Also, the noised image $I_{no}$ needs to be decoded by the Decoder to extract the hidden copyright information, and the extracted copyright information is compared with the original copyright information for accurate extraction, where the loss function is $L_{m}$. The whole model is trained adversarially to make the overall loss of the four loss functions minimized, and then the qualified watermarked image with dual protection of privacy and copyright is generated.

During the adversarial training process, the encoder E and decoder D enable the hiding and extraction of copyright information, thereby fulfilling the function of confirming copyright protection. To improve the image quality and invisibility of adversarial examples, adversarial training is conducted with the discriminator A, allowing the generated adversarial examples to mislead the discriminator’s classification. Meanwhile, a channel attention mechanism module is introduced into the encoder and decoder. This module can automatically learn the weights between channels, effectively focus on important regions in the image, enhance the ability to perceive information in these regions, and also improve the robustness of the trained model. Through continuous interaction (game) with the target classifier T, the generated copyright-verifiable adversarial examples can mislead the classifier, thus possessing attack performance.

### 3.2. Encoder

**Figure 2:**
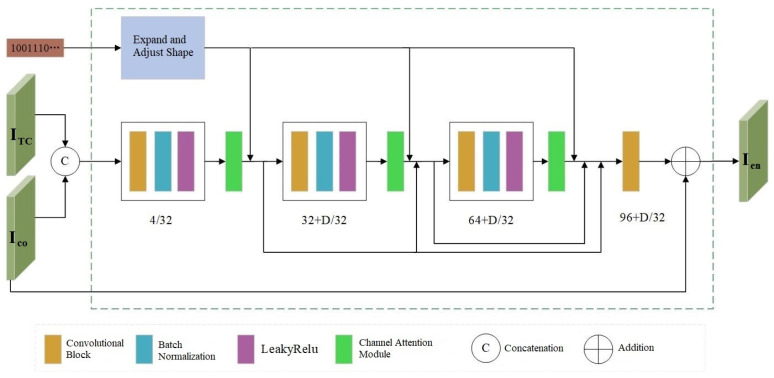
Encoder Structure.

In the framework of encoder E, we take a color cover image $I_{co}\in {\left\{0,\ldots ,255\right\}}^{C\times H\times W}$ with C channels and an image size of H×W, a threshold image $I_{TC}\in {\left\{0,\ldots ,255\right\}}^{1\times H\times W}$, and binary copyright information $M\in {\left\{0,1\right\}}^{L}$ with a length of L as inputs. The copyright information is embedded into the cover image to generate an adversarial example $I_{en}$ containing the copyright information, which can be expressed by Formula (8):

(8) $$I_{en}=E(I_{co},I_{TC},M;\theta_{E}),$$

where $\theta_{E}$ denotes the parameters of the encoder. The threshold image is processed using the Otsu-thresholding method [29], where smooth regions (areas with continuous and uniform pixel values) in the image are assigned a value of 0, and texture regions (areas contain regular or irregular repetitions) are assigned a value of 1. As shown in Figure 2, the encoder adopts a network architecture similar to Dense-Net, consisting of 3 convolutional blocks and 1 convolutional layer. The spatial dimension of the filter in each convolutional block/layer is set to 3 × 3, with both image pixel padding and stride size set to 1. Batch normalization is used to accelerate network training, improve the stability of the model, and reduce the impact of overfitting on the model. The model employs LeakyReLU with a negative slope of 0.01 as the activation function for encoder E. This function can output a non-zero value when the input data is negative, avoiding the phenomenon of neuron death and enhancing the learning ability of the model.

The DenseNet architecture connects each layer with the feature data extracted by all preceding convolutional blocks/layers, ensuring feature reuse and efficient information transmission. It also alleviates the vanishing gradient problem and expands the number of channels in each convolutional block/layer, which helps the network capture more types of features in images, such as edges, colors, and textures. However, in DNNs, convolutional operations typically focus on local spatial information and pay less attention to the relationships between channels. To address this limitation, we incorporate a channel attention module into the network model. This module adaptively assigns different weights to each channel of the image, thereby enhancing the weights of important feature channels and improving the model’s ability to select image features, so as to generate appropriate copyright-verifiable adversarial examples.

### 3.3. Decoder

**Figure 3:**
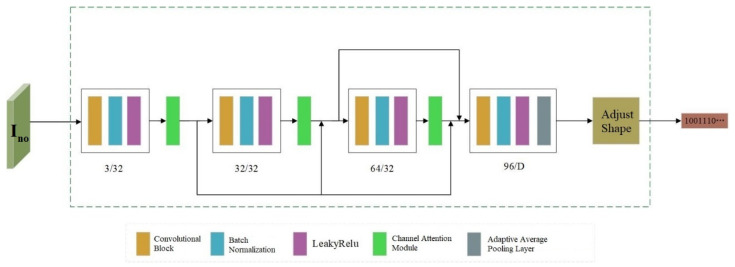
Decoder Structure.

Decoder D takes the adversarial example $I_{no}$ with noise interference as input, and extracts the copyright information $M^{\prime }$ hidden in the image, which can be expressed by Formula (9) as follows:

(9) $$M^{\prime }=D(I_{no};\theta_{D}),$$

where $\theta_{D}$ represents the network parameters of the decoder. By extracting the copyright information, the function of confirming the image copyright is realized. Therefore, the goal of optimizing the decoder is to maximize the accuracy of extracting copyright information. Its network structure is similar to that of Encoder E, as shown in Figure 3. Each convolution block uses Batch Normalization (BN) to standardize the data, which stabilizes the training process of the network model. Meanwhile, LeakyReLU is adopted as the nonlinear activation function of the model, which accelerates the model’s convergence speed and improves training efficiency. To enhance the accuracy of copyright information extraction, channel attention modules are added after the first three convolution blocks of Decoder D respectively, urging the model to learn the embedding positions of copyright information. In the last convolution block, an adaptive average pooling operation is incorporated. This not only reduces the number of model parameters but also scales down the image feature map to a size of 1 × 1 and compresses its shape, so as to restore the original shape of the copyright information.

### 3.4. Discriminator

During the training process, Discriminator A takes the encoded image $I_{en}$ as input to accurately determine whether the input image contains copyright information, which can be expressed by Formula (10).

(10) $$Y/N=D(I_{en}),$$

where Y denotes the input image contains copyright information, and N denotes not.

**Figure 4:**
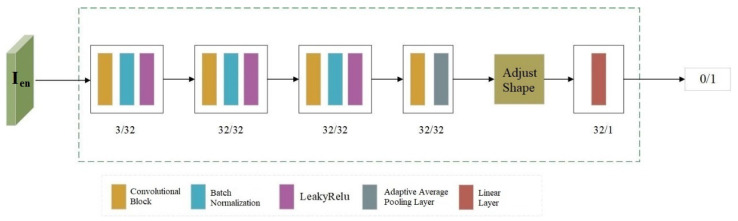
The Structure of Discriminator.

The network structure consists of 4 convolutional blocks. In the last convolutional block, an adaptive average pooling operation is added to reduce the number of feature parameters and modify the feature size. Finally, a linear layer is used for processing to output the judgment result (where 0 represents a cover image and 1 represents an adversarial example with verifiable copyright). The network structure of the discriminator is shown in Figure 4.

During the training process of embedding copyright information, adversarial training with Discriminator A is conducted. This urges Encoder E to continuously optimize its strategies for embedding information and its selection of embedding positions, while also enhancing Discriminator A’s ability to detect the presence of copyright information. Ultimately, this enables the encoder to generate the copyright-embedded images that are more concealed and imperceptible to the human eye.

### 3.5. Target Classifier

We adopt four classification models, i.e., ResNet50, Inception-v3, EfficientNet, and DenseNet121, as attack models. To ensure that the generated adversarial examples can mislead the prediction of the classification models, we introduce pre-trained classification models into the framework. An image $I_{no}$ is input into the pre-trained classification model T, which then outputs a predicted classification label $y_{p}$. As shown in Formula (11).

(11) $$y_{p}=T(I_{no}),$$

We check whether the predicted label $y_{p}$ is consistent with the original label y: if they are inconsistent, it indicates that the image attack is successful and the model has made a misclassification; otherwise, the attack is deemed failed. The parameters of different target attack networks are shown in Table 1.

### 3.6. Loss Function

During the adversarial training process, copyright information is embedded into the cover image, making the generated adversarial examples visually indistinguishable to the human eye. Therefore, it is necessary to ensure that the quality of the generated images does not suffer from severe distortion or similar issues. To address such problems, we use mean squared error (MSE) loss to measure the pixel-level difference between the cover image $I_{\mathrm{co}}$ and the adversarial example $I_{en}$, thereby improving the visual quality of the images. This is shown in Formula (12):

(12) $$L_{g}=\mathrm{MSE}(I_{\mathrm{co}},I_{en})=\frac{1}{C\times W\times H}{\left\|I_{co}-I_{en}\right\|}_{2}^{2}$$

During the process of image copyright verification, we need to use Decoder D to extract the copyright information from images with noise interference. Additionally, it is necessary to minimize the difference between the original copyright information M and the extracted copyright information $M^{\prime }$. Therefore, we use MSE loss to calculate the error between them, which can be expressed as the following Formula (13):

(13) $$L_{m}=\mathrm{MSE}(M,M^{\prime })=\frac{1}{L}{\left\|M-M^{\prime }\right\|}_{2}^{2}$$

For the generation of adversarial examples, Encoder E engages in a “game” with Discriminator A, which drives the copyright information in the generated adversarial examples to be invisible. In this paper, we train Discriminator A not only to determine whether an image contains copyright information, but also to improve the information embedding strategy and image quality. The training goal is to enable the generated adversarial examples to mislead the discriminator’s classification and prompt the binary-class discriminator to output incorrect labels. Therefore, we use the binary cross-entropy adversarial loss function for calculation, which can be expressed as the following Formula (14):

(14) $$L_{adv}=E_{I_{co}}\log(A(I_{co});\theta_{A})+E_{I_{en}}\log(1-A(I_{en});\theta_{A})$$

where *A*(.) denotes the output of Discriminator, and $E_{I_{co}}$ derives with empirical distribution.

To ensure the security of users’ image information from the network source, it is necessary to effectively counter the powerful retrieval and classification capabilities of DNNs, thereby preventing images from being exploited and tampered with. Therefore, we design a type of adversarial example with verifiable copyright that features image copyright protection functionality. The goal is to maximize the difference between the original label and the predicted label, thereby misleading the output of the target classifier T and causing the classifier to output incorrect labels. This can be expressed as the following Formula (15):

(15) $$L_{cla}=Tp(y|I_{no})-\max\{Tp(i|I_{no}):i\neq y,\;\;$$

where y represents the true label of image $I_{no}$, and $Tp(i|I_{no})$ represents the probability that the predicted label of image $I_{no}$ is i. The overall goal of training the entire network framework is to minimize the total loss function, so that the images embedded with binary copyright information can meet requirements in four aspects: image quality, invisibility of copyright information, information extraction, and adversarial attack. The total loss function can be expressed as the following Formula (16):

(16) $$L=\mathrm{Minimize}\;\lambda_{g}L_{g}+\lambda_{cla}L_{cla}+\lambda_{m}L_{m}+\lambda_{\mathrm{adv}}L_{adv},$$

where $\lambda_{g}$, $\lambda_{cla}$, $\lambda_{m}$ and $\lambda_{\mathrm{adv}}$ represent the weights indicating the importance of each loss respectively.

## 4. Experiments

### 4.1. Datasets and Experimental Setup

To test the effectiveness of the proposed model, we conducted experiments on the PyTorch 1.12 platform using an NVIDIA GeForce RTX 3090 GPU, with training and testing performed on the Caltech256 dataset. Specifically, 24,000 images were randomly selected from the dataset as the training set, and 3000 images were chosen as the test set. During the training process, we first performed image preprocessing operations, including scaling, rotation, and normalization of the images—these steps were implemented to increase data diversity and enhance the robustness of the model. The embedded binary copyright information was sampled from a Bernoulli distribution with a probability of 0.5. The model was trained for 40 epochs, and the Adam optimization algorithm with a learning rate of 2 × 10^−4^ and a learning rate decay strategy were adopted to improve the model’s training efficiency. For the weight parameters of the model’s training loss function, we set $\lambda_{g}$ = 1000, $\lambda_{cla}$ = 10, $\lambda_{m}$ = 100, and $\lambda_{\mathrm{adv}}$ = 1, and the intensity factor was set to 1. The parameters are chosen by balancing image quality, invisibility of copyright information, information extraction, and adversarial attack through thorough experiments.

To verify the effectiveness of the model’s noise-resistant training, we introduced six types of noise for training, namely Crop, Cropout, Dropout, JPEG compression, Gaussian blur, and Resize. We adjusted the parameters of the noise to observe their interference with the model. In addition, we compared our model with the watermarking models HiDDeN [14], MBRS [16], TSDL [15], and IAW [22]. To ensure a fair comparison, the information length of all models was set to 60.

### 4.2. Evaluation Metrics

(1) Attack Success Rate (ASR)

A DNN maps an input image to a vector, where this vector represents the probability distribution or confidence level of the image belonging to different categories. The category with the highest confidence level is taken as the predicted category. When an adversarial example is input into the attack model, if the output result is inconsistent with the true result of the cover image, it indicates that the adversarial example has successfully attacked the model. The attack success rate is calculated by dividing the number of times adversarial examples successfully deceive the model by the total number of adversarial examples used in the test. The calculation method is shown in the Formula (17):

(17) $$\mathrm{ASR}=\frac{\sum_{i=1}^{n}I\left(T(X^{\left(i\right)})=y^{\left(i\right)}\wedge T(X^{(i)\prime })\neq y^{\left(i\right)}\right)}{\sum_{i=1}^{n}I\left(T(X^{\left(i\right)})=y^{\left(i\right)}\right)}\times 100\%,$$

where *T*(.) denotes the output label of the classifier *T*, the numerator of the formula represents the number of successfully attacked examples, the denominator represents the total number of image examples, and ∧ denotes the logical AND operation. The value range of ASR is [0, 1]; a higher value indicates a better attack effect of the images.

(2) Peak Signal-to-Noise Ratio (PSNR)

We introduce PSNR as a metric to measure the similarity between the cover image and the generated adversarial example. The difference between each pixel value of the C×H×W-sized cover image X and the adversarial example $X^{\prime }$ is calculated using MSE, and the calculation of MSE is shown in Formula (18).

(18) $$\mathrm{MSE}=\frac{1}{C\times H\times W}\sum_{i=1}^{C}\sum_{j=1}^{H}\sum_{k=1}^{W}(X_{i,j,k}-{X^{\prime }}_{i,j,k}{)}^{2}$$

And PSNR can be calculated by Formula (19):

(19) $$\mathrm{PSNR}=10\times \log_{10}(\frac{MAX^{2}}{MSE}),\;$$

where MSE represents the maximum possible range of image pixel values. In the lossy compression of images, the typical value of PSNR ranges from 30 to 50 dB; a larger value indicates a higher similarity between the lossy image and the cover image, while a smaller value, by contrast, means the lossy image suffers from more severe distortion.

(3) Structural Similarity (SSIM)

SSIM is mainly used as an indicator to detect the similarity between two images of the same size or to measure the distortion degree of an image. It takes into account three key features of the two images: Luminance, Contrast, and Structure. This is shown in Formula (20):

(20) $$\mathrm{SSIM}\left(x,y\right)=\frac{\left(2\mu_{x}\mu_{y}+C_{1})(2\sigma_{xy}+C_{2}\right)}{\left(\mu_{x}^{2}+\mu_{y}^{2}+C_{1})(\mu_{x}^{2}+\mu_{y}^{2}+C_{2}\right)}$$

where $\mu_{x}$, $\mu_{y}$, $\mu_{x}^{2}$, $\mu_{y}^{2}$ respectively represent the mean values and variances of images x and y, $\sigma_{xy}$ denotes the covariance of x and y, and $C_{1}={\left(k_{1}L\right)}^{2}$ and $C_{2}={\left(k_{2}L\right)}^{2}$ are constants used to maintain the stability of the fraction, preventing the denominator from being zero. L=(0,255) represents the range of image pixel values, and by default, $k_{1}$ = 0.01 and $k_{2}$ = 0.03 are used. The value range of SSIM is from 0 to 1; a larger value indicates a higher similarity between the two images. When the two images are identical, the value of SSIM is 1.

(4) Extraction Accuracy (ACC)

Useful data is embedded into the cover image to generate adversarial examples; meanwhile, the data needs to be extracted, and a high extraction accuracy must be ensured. Since the range of data extracted by the network is [0, 1], the extracted data is rounded to {0, 1}. The extraction accuracy is calculated by dividing the sum of the bits where the extracted ciphertext $M^{\prime }$ is equal to the original ciphertext M by the total number of bits of the ciphertext. The calculation method is shown in Formula (21):

(21) $$\mathrm{ACC}=\frac{\sum_{i=1}^{N}I\left(M_{i}=M_{i}^{\prime }\right)}{N}\times 100\%,\;$$

where I is a logical indicator function, which outputs 1 when the ciphertext M is equal to the extracted ciphertext $M^{\prime }$. A higher accuracy indicates that the extracted ciphertext is closer to the original ciphertext. Conversely, the Bit Error Rate (BER) refers to the probability of errors in the extracted information, and its calculation method is shown in the Formula (22):

(22) BER=1−ACC

### 4.3. Adversarial Attack Capability

To verify the attack capability of the adversarial examples generated by the proposed model, we conducted experiments on the ResNet50 classifier using images generated by different watermarking models (HiDDeN [14], TSDL [15], MBRS [16]) and images generated by our designed model. Meanwhile, we compared the average results of the images generated by these different models.

**Figure 5:**
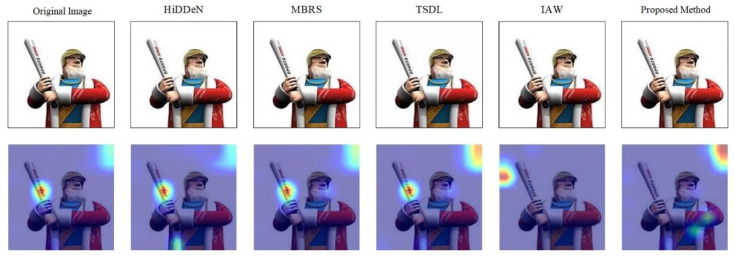
Grad-CAM Visualization Heatmaps of Different Watermarking Models and the Proposed Method on ResNet50.

The experimental results are shown in Table 2. We found that when the adversarial attack performance of images is not considered, the ASR results of the HiDDeN, TSDL, and MBRS methods are very small. This also indicates that these three methods only embed information into images in a hidden manner and retain the main semantics and features of the images in most cases, resulting in poor attack effects. At the same time, the IAW method exhibits excellent attack capability, which proves that both the IAW method and our proposed method have affected the underlying features of images. This causes the classification model to have deviations or errors in understanding high-level semantics, leading to a high attack success rate. Figure 5 shows the Gradient-weighted Class Activation Mapping (Grad-CAM) visualization heatmaps of images generated by different models under the same classifier. The heat map converts abstract data into a visually perceptible “temperature field,” and reflects the attention information density through color gradients (from red to blue). Compared with the results of other watermarked images, our method has significantly changed the attention areas of image classification decisions in the heatmaps. This indicates that the copyright-verifiable adversarial examples generated by our model have affected the activation of intermediate layers in the neural network classifier, making the network focus on non-critical or irrelevant areas in the images. As a result, the highlighted areas of Grad-CAM deviate from the feature areas that are crucial for the correct original classification of the images.

### 4.4. Generating Adversarial Examples in Stages

Since there are relatively few research methods on adversarial examples with copyright protection functions, and to increase the experimental control, we conducted phased experiments by combining popular deep learning-based watermarked image generation methods (HiDDeN [14], TSDL [15], and MBRS [16]) with adversarial attack methods (AdvGAN [5], C&W [4], FGSM [3], and PGD [6]). Finally, we generated adversarial examples containing copyright information and compared them with the model we proposed.

During the experiment of generating adversarial examples in stages, for the process where adversarial attack experiments are conducted first, followed by information embedding to generate adversarial examples, the information embedding in the second stage may destroy the key adversarial information in the adversarial examples from the first stage. This weakens or eliminates the network’s misjudgment effect on the target category, thereby reducing the effectiveness of the attack and ultimately affecting the attack success rate of the generated adversarial examples. Meanwhile, the results of this experiment have also been verified in IAW [22]. Therefore, we only consider the comparison of another phased experiment, i.e., first performing information embedding and then conducting the adversarial attack. 

**Figure 6:**
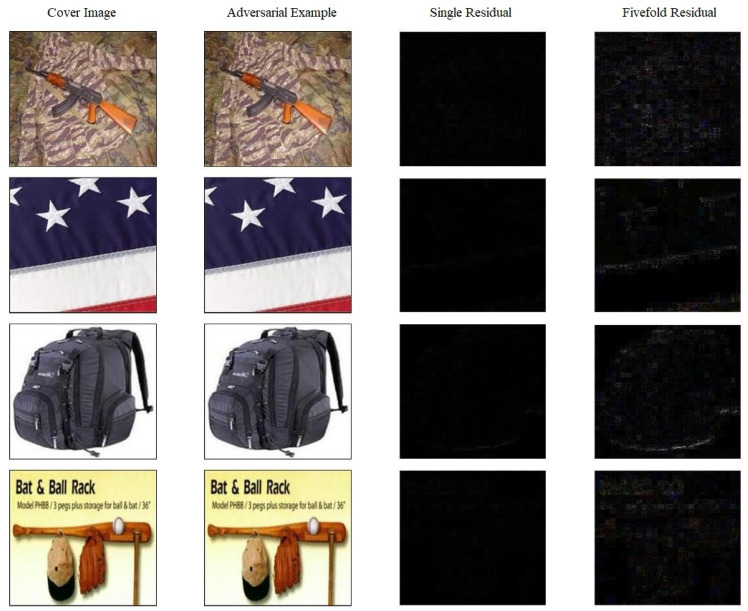
Display of Image Results on the Test Dataset. From left to right: cover image, adversarial example, single residual and fivefold residual between the cover image and the adversarial example. Average image quality: PSNR = 37.92, ASR = 98.83%, BER = 0%.

The relevant experimental results are shown in Table 3, where the bold numbers denote the best results. Some works achieved a relatively good ASR (e.g., 100%). However, they also led to a higher Bit Error Rate (BER) compared with our results. In other cases, for example, in the experiment combining TSDL and FGSM, the BER values obtained across the four classifiers were all greater than 40, making it impossible to extract information accurately. Compared with these experiments, the ASR of our method in experiments on different classifiers all exceeded 98%, and the BER was very close to 0. Additionally, the quality of the generated adversarial example images was also the best in comparison, as shown in Figure 6, where “Single residual” represents the original residual without any processing, while “fivefold residual” refers to the original residual scaled up by a factor of five to facilitate easier observation. Therefore, the adversarial examples generated by the proposed network structure exhibit extremely excellent performance in both information extraction and adversarial attacks.

### 4.5. Robustness Test

To verify the robustness of the copyright-verifiable adversarial example model under different environments, we trained the model using different noise types separately: Crop, Cropout, JPEG, Dropout, Gaussian blur, and Resize, with different noise parameters set. For the model trained with specific noise, we refer to this specific model as “Specified”. Meanwhile, we also trained a model by randomly combining different noise layers, which we call “Combined”. For the model not trained with any noise, we term it “Identity”. To measure the model’s robustness more accurately and reduce interference from other factors, we set the image quality metric PSNR to 35.00 ± 0.1 and designated the target classifier as ResNet50.

**Figure 7:**
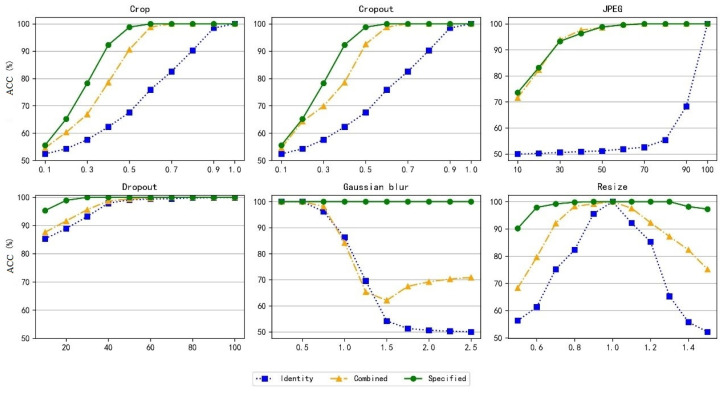
ACC of Information Extraction Under Different Noise Environments.

The experimental results are shown in Figure 7. It can be observed that under most circumstances, the proposed method achieves favorable results. However, the Identity model, which was not trained with noise, performs poorly. For instance, after JPEG compression, the accuracy of information extraction decreases significantly and mostly remains around 50%, which means that after compression processing, the extracted information is similar to random guessing. In contrast, the Specified model achieves excellent performance after experiments with different noise parameters. For example, under the noise conditions of Crop (0.5), Cropout (0.5), and JPEG (50), the model’s information extraction accuracy reaches over 98.5%. Meanwhile, the extraction accuracy of the Combined model, which was trained with random noise layers, is also superior to that of the Identity model. This indicates that noise layers can guide the model to learn and maintain robustness when images are distorted.

**Figure 8:**
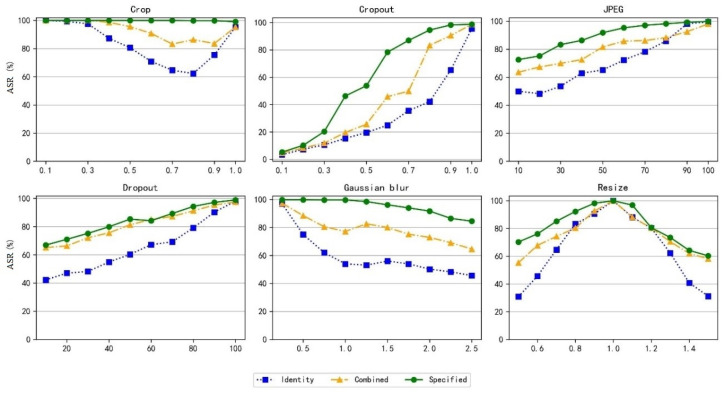
ASR Under Different Noise Environments.

Meanwhile, we also verified the robustness of the model’s adversarial attack capability under different environments, and the experimental results are shown in Figure 8. It can be observed that under different noise environments, the ASR of the model trained with specific noise is significantly superior to that of the model not trained with noise. This not only verifies that training with noise layers can improve the model’s robustness and security under different noise environments, but also indicates that the proposed model has strong robust characteristics and can adapt to different noise environments, thereby achieving the effect of protecting image privacy.

### 4.6. Ablation Experiment

To verify the effect of the channel attention module on the adversarial example generation model, we conducted an ablation experiment. With other network models and parameters unchanged, we removed the attention mechanism modules from the encoder and decoder, set the watermark length to 60, and tested the PSNR, ASR, and ACC results of the ablated experiment and the original model.

The experimental results are shown in Table 4. It can be observed that the model with the channel attention mechanism added outperforms the model without the attention mechanism in all comparative experimental metrics. This verifies that the channel attention module can enhance the attack capability of adversarial examples and utilize the correlation between image channels to embed data into appropriate channels, thereby adjusting the impact of embedded data on images.

## 5. Conclusions

In this paper, by leveraging the characteristics of CGAN and channel attention mechanisms, and integrating the copyright protection function of watermarks with the attack and invisibility properties of adversarial examples, a network architecture for CCIW is constructed. The encoder combines copyright information, threshold images, and cover images to generate adversarial examples containing copyright information, while the decoder extracts the corresponding copyright information from these examples to achieve copyright confirmation and protection of images. Simultaneously, adversarial training is conducted with a well-trained target classifier to deceive the classifier, thereby realizing adversarial attacks against malicious network collection or infringement behaviors that utilize DNNs. Through experimental analysis under different scenarios, we verified the advantages of this model in terms of extraction capability, attack capability, and robustness, and proved the effectiveness of this method in protecting the copyright and privacy of images. The proposed method can be used in various applications, such as social media platforms and digital content distribution, since they all face privacy threats where attackers use DNNs for search and recognition in addition to copyright threats, and therefore dual protection of privacy and copyright is needed.

## Figures and Tables

**Table 1:** Parameters of Different Target Attack Networks.

Network Model	Accuracy	Number of Parameters
ResNet50	85.76%	24.0 M
Inception-v3	82.53%	23.8 M
EfficientNet	84.03%	18.0 M
DenseNet121	83.01%	72.1 M

**Table 2:** ASRs of Different Models Tested on the ResNet50 Classifier.

Model	HiDDeN (%)	TSDL (%)	MBRS (%)	IAW (%)	Proposed (%)
ASR	6.62	2.29	2.20	98.20	98.52

**Table 3:** Comparison of Generating Adversarial Examples in Stages.

Stage 1	Stage 2	ResNet-50			DenseNet-121			VGG16			Inception-v3		
		BER (%)	PSNR (dB)	ASR (%)	BER (%)	PSNR (dB)	ASR (%)	BER (%)	PSNR (dB)	ASR (%)	BER (%)	PSNR (dB)	ASR (%)
HiDDeN	AdvGAN	2.73	32.68	97.69	0.56	33.33	99.20	3.04	30.25	98.27	3.35	31.73	97.57
	C&W	0.24	33.79	100	0.22	33.80	**100**	0.25	33.77	**100**	0.21	33.77	**100**
	FGSM	3.55	30.63	77.44	3.53	30.69	89.22	3.66	29.93	96.46	3.69	30.41	63.61
	PGD	3.26	31.88	**100**	3.21	31.93	**100**	3.38	31.32	**100**	3.34	31.94	98.92
TSDL	AdvGAN	5.17	34.28	97.95	2.52	35.47	99.26	3.74	31.12	97.98	3.16	33.08	97.04
	C&W	1.66	33.59	**100**	1.77	33.56	**100**	1.98	33.59	**100**	1.75	33.55	**100**
	FGSM	44.89	30.52	75.38	43.8	30.56	88.97	44.58	29.84	96.76	45.10	30.32	63.07
	PGD	42.31	31.75	**100**	42.0	31.80	**100**	43.56	31.22	**100**	42.88	31.78	99.19
MBRS	AdvGAN	0.11	34.62	97.69	0.51	35.98	99.26	0.04	31.33	97.98	4.687	33.32	96.77
	C&W	0.54	35.62	**100**	0.62	36.68	98.47	0.57	35.36	**100**	0.653	34.52	**100**
	FGSM	4.50	31.77	73.01	4.76	31.84	88.75	0.009	30.88	94.97	5.484	31.49	63.44
	PGD	2.32	33.51	**100**	2.169	33.54	**100**	2.185	32.68	**100**	2.493	33.53	99.21
IAW		**0**	36.12	98.20	0.001	38.00	98.77	**0**	35.40	99.12	**0**	34.54	98.00
Proposed Method		**0**	**37.45**	98.16	**0**	**38.86**	98.25	0.002	**37.54**	98.94	**0**	**36.86**	98.23

**Table 4:** Comparison of Ablation Experiment.

Index	Proposed Method	Ablation Experiment
PSNR	37.5	30.8
ASR	98.2%	85.6%
ACC	99.82%	97.15%

## Data Availability

The datasets generated during the current study are available from the corresponding author on reasonable request.
